# In Vitro Synergy of Levofloxacin Plus Piperacillin/Tazobactam against *Pseudomonas aeruginosa*

**DOI:** 10.1155/2009/984934

**Published:** 2009-11-08

**Authors:** Vladimir Chachanidze, Aixa Curbelo-Irizarry, Deborah Ashcraft, George Pankey

**Affiliations:** ^1^Ochsner Clinic Foundation, 1514 Jefferson Highway, New Orleans, LA 70121, USA; ^2^Premier Infectious Disease Care, 320 Edinburgh Dr., Winter Park, FL 32792, USA

## Abstract

In vitro synergy testing using levofloxacin (LVX) plus piperacillin/tazobactam (TZP) was performed by Etest and time-kill assay (TKA) for 31 unique fluoroquinolone-resistant *Pseudomonas aeruginosa* isolates. The Etest method showed synergy for 9/31 (29%) of isolates, while TKA showed synergy with 14/31 (45%) of isolates. When comparing the Etest method and TKA, concordant results for synergy, antagonism, and indifference were obtained for 24/31 (77%) of the isolates tested.

## 1. Introduction

*Pseudomonas aeruginosa* is a major nosocomial pathogen, and effective therapy represents a great challenge. *P. aeruginosa* strains are often resistant to antibacterial agents from different classes, including *β*-lactams, aminoglycosides, and fluoroquinolones; some strains are only susceptible to polymyxins [1]. The mechanisms of resistance of *P. aeruginosa* are determined by both chromosomal and plasmid genes encoding different resistance enzymes (*β*-lactamases, including extended spectrum *β*-lactamases, carbapenemases, etc.); other mechanisms include decreased bacterial wall permeability, target alterations, and active drug efflux [2].

Commonly, treatment of *P. aeruginosa* involves a combination of antibacterial agents. Levofloxacin (LVX) plus piperacillin/tazobactam (TZP) is an antipseudomonal regimen used in many hospitals. The putative benefits are to increase efficacy by achieving synergistic killing and preventing the emergence of antibiotic resistance, but data are sparse. A recent retrospective cohort study demonstrated a reduction in 28-day all-cause mortality in less severely ill patients (533 of 702 patients, (75.9%)) with monomicrobial bacteremia due to aerobic gram-negative bacilli (including *P. aeruginosa*) who received either a combination of a *β*-lactam plus LVX or ciprofloxacin versus *β*-lactam monotherapy [3]. This supported an in vivo synergistic or additive effect of the *β*-lactam plus fluoroquinolone combination.

Three methods to detect in vitro synergy have been described: the time-kill assay (TKA), checkerboard, and Etest method. Earlier in vitro studies using TKA and checkerboard techniques suggested different rates of synergy (17–83%) of LVX plus TZP (4–6; H Jones and E Swiatlo, 41st Annual Meeting Infectious Diseases Society of America, Abstr. 230, 2003) or piperacillin [4–6] against *P. aeruginosa*. Two TKA studies by Burgess et al. [7, 8] were similar with 12 *P. aeruginosa* isolates and showed 67–83% synergy. However, only 4/12 isolates were resistant to either LVX or TZP. Another study—by Jones and Swiatlo [2003]—with the same combination tested 100 *P. aeruginosa* isolates by the checkerboard method, showing 17% synergy. Drago et al. [9] performed the checkerboard and TKA methods against resistant *P. aeruginosa*, showing synergy in 6/30 (20%) and (75%) isolates, respectively. Studies by White et al. [10] and Bonapace et al. [11] evaluated the use of Etest for synergy testing by placing the Etest strips on the agar in a cross formation, with a 90° angle at the intersection between the scales at the respective MICs for the organism. In the study by White et al. [10], the agreement between their Etest method and TKA ranged 63–75% and agreement between the checkerboard method and TKA ranged 44–88%. Correlation was dependent on the bacterium (*Escherichia coli *ATCC 35218, *Enterobacter cloacae *ATCC 23355, *P. aeruginosa *ATCC 27853, and *Staphylococcus aureus *ATCC 29213) and antibiotic tested (cefepime or ceftazidime in combination with tobramycin or ciprofloxacin). Similarly, in the study by Bonapace et al. [11], the agreement between Etest and TKA ranged 42–97% and 30–67% for the checkerboard method and TKA, respectively. This study included 10 strains of *Acinetobacter baumannii*, and antimicrobial combinations evaluated consisted of trovafloxacin or tobramycin in combination with cefepime or piperacillin. Antagonism was difficult to detect with their method. Both studies concluded that additional testing using an Etest method needed to be performed.

Synergy testing methods are not standardized for reproducibility and interpretation; therefore, it is extremely difficult to compare these methods' results from different studies. In the TKA for synergy, drug concentrations are fixed and do not decrease over time, as they would in vivo. Also, there are no standard concentrations at which antibiotics are tested. The inoculum size and time frame of the TKA add more variability to the test. The time parameter of 24 hours can limit or alter results of the experiment if regrowth occurs with one or both antibiotics. Regrowth can be caused by the use of a subinhibitory concentration of antibiotics. Emergence of resistant subpopulations may account for the regrowth, or regrowth may be due to bacteria that adhere to the surface of the bottle and are subsequently released in the medium. Another factor affecting regrowth is inactivation of the antibiotics in vitro. The TKA for synergy testing measures bactericidal activity but is time-consuming and labor-intensive.

In the checkerboard technique, serial dilutions of two drugs are performed in tubes or microtiter wells using drug concentrations equal to, above, and below the MICs of the drugs being tested. The checkerboard method measures inhibitory activity. Only if each microdilution well at the MIC and greater is subcultured for growth would this method predict bactericidal activity. Because TKA and checkerboard measure different activities, study results have shown poor agreement [10–14]. There are limitations associated with both methods. In the study by Cappelletty and Rybak [13], methodologies for synergy testing of resistant *P. aeruginosa *were compared, and problems were discussed.

The third method for determining synergy, the Etest synergy method, is relatively new. The use of the Etest strip for synergy has yet to be standardized but has the potential to be a useful screening test for determining synergy. We have further modified the Etest synergy method to use a concentration equal to 1 × MIC for each drug [15]. An MIC-to-MIC placement of the strips seems to give a more accurate diffusion of the two drugs and the effects (if any) that each drug has on the other in combination against the organism. The technique is simple to use, time-efficient, and inexpensive. Because the checkerboard method and TKA are laborious and time-consuming [16], they are not performed in clinical laboratories. With no approved standard method for in vitro synergy testing available, Etest could be an alternative method for the study of the activities of antimicrobial combinations.

With the recently published clinical study by Al-Hasan et al. [3] showing in vivo synergy with the commonly used antipseudomonal LVX/TZP combination, we felt that adding more in vitro synergy data, including 31 highly-resistant *P. aeruginosa* strains, was indicated. The aim of this present study was twofold: (1) to test for synergistic activity of LVX and TZP against *P. aeruginosa, *including fluoroquinolone-resistant strains and (2) to compare results from two different synergy testing methods, TKA and Etest.

## 2. Materials and Methods

Thirty-one unique clinical, genetically distinct fluoroquinolone-resistant *P. aeruginosa* isolates were collected from October 1999 through June 2003 from five hospitals in the New Orleans area. Fingerprinting of isolates was performed by pulsed-field gel electrophoresis, using criteria by Tenover et al. [17], where a 0–3 band difference is interpreted as indistinguishable. The isolates were cultured from the lower respiratory tract (11), wound (9), urine (6), blood (2), ear (1), catheter tip (1), and bone (1). All strains' identification and susceptibility testing were performed by the Vitek System (bioMérieux Inc., Hazelwood, MO, USA). The percent susceptible was amikacin (58%), ceftazidime (29%), ceftriaxone (10%), gentamicin (16%), imipenem (35%), TZP (39%), LVX (0%), ciprofloxacin (0%), and tobramycin (74%). *P. aeruginosa* ATCC 27853 was used for quality control. Mueller-Hinton broth (Becton-Dickinson Microbiology Systems, Sparks, MD, USA) was prepared in the laboratory. Mueller-Hinton II agar (MHA) plates (Becton-Dickinson) were used for the Etest MIC determination and the Etest synergy method. Trypticase soy agar with 5% sheep blood plates (Becton-Dickinson) were used for the colony counts in TKA. Standard laboratory powders—LVX (Johnson and Johnson Pharmaceutical Research and Development, Springhouse, PA, USA) and TZP (Wyeth Research, Pearl River, NY, USA)—and Etest strips (AB Biodisk, Solna, Sweden) were used.

 Etest MICs for LVX and TZP were determined in triplicate following the manufacturer's guidelines and the mean used. The Etest concentration range (*μ*g/mL) tested was 0.002–32 for LVX and 0.016–256 for TZP. Clinical and Laboratory Standards Institute (CLSI) interpretive standards (*μ*g/mL) for *P. aeruginosa *were applied: LVX ≤ 2 is susceptible, = 4 intermediate, ≥ 8 resistant; TZP ≤ 64 is susceptible and ≥ 128 is resistant [18].

### 2.1. Synergy Testing

#### 2.1.1. Etest Synergy Method

The Etest synergy method [15] was performed in triplicate, the summation fractional inhibitory concentration (∑FIC) was calculated for each set of MICs, and the mean *∑*FIC was used for comparison to the TKA results. The inoculum and streaked MHA plates for each isolate were prepared the same as for Etest MICs. LVX and TZP Etest strips were applied to different sections of an MHA plate. The agar was marked adjacent to the previously determined MIC on each Etest strip. The strips were removed after incubating for 1 hour at room temperature. Using an Etest applicator, a new LVX strip was placed over the area of the previously removed TZP strip so that the LVX MIC corresponded with the mark of the TZP MIC. TZP strips were applied in reciprocate fashion. This established a concentration ratio of 1 × MIC for each of the two antimicrobials. The resulting combination ellipses were read after approximately 20 hours of incubation at 35°C. To evaluate the effect of the combinations, the FIC was calculated for each antibiotic in each combination, and the mean *∑*FIC was used for comparison to TKA. High off-scale MICs were converted to the next twofold dilution. The following formulas were used to calculate ΣFIC: (1) FIC of LVX = MIC of LVX in combination/MIC of LVX alone; (2) FIC of TZP = MIC of TZP in combination/MIC of TZP alone; (3) ΣFIC = FIC of LVX + FIC of TZP. Synergy was defined as ΣFIC ≤ 0.5. Antagonism was defined as ΣFIC > 4. Interactions with ΣFIC > 0.5 but ≤ 4 were termed indifferent [16].

#### 2.1.2. Time-Kill Assay

TKAs were performed according to CLSI guidelines [19]. An approximately 10^5^ CFU/mL inoculum was verified after plating in duplicate using a spiral plater and scanner (Spiral Biotech, Bethesda, MD, USA). Each isolate was tested against LVX and TZP alone and in combination at a concentration equal to the mean Etest MIC. A concentration equal to the mean Etest MIC was used so that TKA results could be compared directly with the Etest synergy method (which uses 1 × MIC for each drug). When the MIC for either drug was greater than the highest concentration value on the strip, the highest concentration on the strip was used in the TKA. Colony counts on all isolates were performed at 0 hour and 24 hours. Performing serial dilutions and plating with a spiral plater, which further dilutes and plates the sample, helped reduce the possibility of antibiotic carryover. The spiral plater/scanner was used to accurately detect bacterial counts as low as 20 CFU/mL.

Synergy was defined as a ≥ 2 log_10_ decrease in colony count after 24 hours by the combination compared to the most active single agent, and the number of surviving organisms in the presence of the combination had to be ≥ 2 log_10_ CFU/mL below the starting inoculum [16]. Indifference was defined as a < 2 log_10_ increase or decrease in colony count at 24 hours by the combination compared to that by the most active drug alone. Antagonism was defined as a ≥ 2 log_10_ increase in colony count after 24 hours by the combination compared to the most active drug alone [16]. TKA results, which were discordant to the Etest synergy results, were repeated and confirmed the initial TKA findings.

## 3. Results and Discussion

Etest MICs for the *P. aeruginosa* isolates were LVX 8- >32 *μ*g/mL (all resistant) and TZP 4- >256 *μ*g/mL (61% resistant). Synergy was found in 9/31 (29%) of isolates using Etest and 14/31 (45%) using TKA. Six isolates were indifferent by the Etest method (∑FIC: 2, 1, 2, 0.9, 0.8, 1) but synergistic with the TKA method (log_10_ change: −2, −2, −3.2, −4, −3.8, −3.3). One isolate was synergistic with Etest (∑FIC = 0.4) but indifferent with TKA (log_10_ change = 0). There was good agreement for synergy between Etest (32%) and TKA (37%) when both drugs were resistant. Agreement was not as good between Etest and TKA (25% vs. 58%) when TZP was susceptible Concordance between Etest and TKA was high: 24/31 (77%) (see Table 1) However since there no gold standard for synergy testing it is difficult to establish which method is more accurate. There was no evidence of in vitro antagonism.

At least three possible reasons may explain the discrepancies in results between Etest and TKA. First, our isolates were highly LVX (100%) and TZP (61%) resistant, with MICs often exceeding the Etest strip detection limit; therefore, we empirically used the next twofold dilution for ΣFIC calculation. This makes it difficult to interpret the results and is clearly a limitation of the Etest method. Second, the current Etest ΣFIC criteria for synergy are simply imported from the checkerboard methodology and may need adjustments for Etest. Last, the TKA data were analyzed based on CFU/mL changes comparing the effect of the combination with the effect of the most potent drug, whereas Etest data were analyzed with FIC indexes comparing concentrations of the drugs alone and in combination.

Etest was able to detect slight hazes of growth and resistant subpopulations. Because the MICs of both drugs were read as bactericidal endpoints, these resistant colonies were included when reading the endpoint for the Etest MIC. Lorian showed that bacteria grown on a surface are significantly different from bacteria grown in liquid medium [20]. The differences include growth rate, adherence, and susceptibility to antibacterial agents, as well as differences in the biochemical constitutions of the bacteria themselves and their metabolites. One major difference is in the ultrastructure. Evidence indicates that bacteria in vivo grow and produce disease on surfaces and not in body fluids [20–23]. The identical ultrastructures of bacteria found in vivo and organisms grown in vitro on a surface support the theory that in vitro experiments aiming at duplicating in vivo conditions should be performed on solid media.

The present study, which included all LVX-resistant and 61% TZP-resistant *P. aeruginosa *strains, showed synergy by Etest (29%) and TKA (45%) and confirms previous in vitro synergy data using *β*-lactam/fluoroquinolone combinations. Previous in vitro synergy studies showed 17–83% synergy, depending on the method used.

## 4. Conclusion

Some in vitro synergy with LVX plus TZP could be demonstrated with both TZP-susceptible and TZP-resistant *P. aeruginosa* strains, and no antagonism was found. The high concordance (77%) between Etest and TKA suggests that the Etest method may be an alternative to time-kill studies for in vitro synergy testing of *P. aeruginosa *with LVX and TZP. Etest is simple to use, time-efficient, inexpensive, and reproducible and yielded results comparable to TKA. In vitro synergy may or may not translate into in vivo synergy.

## Figures and Tables

**Table 1:** Etest MIC (*μ*g/mL), Etest Synergy Method, and Time-Kill Assay.

*P. aeruginosa*				
*n* = 31	LVX MIC	TZP MIC	Synergy Testing (LVX + TZP)	

			Etest	TKA
	Etest^(a)^	Etest^(a)^	ΣFIC^(a)^	Log_10_change^(b)^

1	> 32	> 256	2.0 I	−1.4 I
2	8	> 256	0.4 S	−3 S
3	32	> 256	0.4 S	0 I*
4	> 32	> 256	2.0 I	−2 S*
5	> 32	4	1.2 I	−0.7 I
6	> 32	16	0.5 S	−3.4 S
7	> 32	4	1.0 I	−2 S*
8	> 32	> 64	2.0 I	+1.3 I
9	32	> 4	0.004 S	−2.4 S
10	> 32	> 4	0.8 I	+1.4 I
11	> 32	> 256	1.5 I	+1.3 I
12	> 32	> 256	2.0 I	−1.5 I
13	> 32	> 256	1.2 I	−0.7 I
14	> 32	> 256	2.0 I	−0.4 I
15	> 32	> 256	2.0 I	−1 I
16	> 32	> 256	0.2 S	−2 S
17	> 32	16	1.4 I	−0.4 I
18	> 32	> 256	2.0 I	−0.6 I
19	> 32	> 256	1.3 I	−1.1 I
20	> 32	> 256	2.0 I	−3.2 S*
21	> 32	32	0.9 I	−4S*
22	> 32	> 256	2.0 I	−0.1 I
23	> 32	32	0.5 S	−2 S
24	> 32	16	1.1 I	−0.4 I
25	> 32	> 256	0.6 I	−1.3 I
26	> 32	32	0.8 I	−3.8 S*
27	> 32	> 256	0.5 S	−2 S
28	> 32	8	1.0 I	−3.3 S*
29	> 32	> 256	1.1 I	−1.1 I
30	> 32	> 256	0.3 S	−2.1 S
31	> 32	> 256	0.3 S	−2.8 S

*Pseudomonas aeruginosa strains with discordant synergy results. ^(a)^Performed in triplicate. ^(b)^Values represent the log_10_ change in CFU/mL in the time-kill assay after 24 hour exposure to the combination of levofloxacin (LVX) and piperacillin/tazobactam (TZP) compared to the most active drug alone. Negative values indicate a decrease in colony count; positive values indicate an increase in colony count. S: synergy; I: indifference.
